# Studying the effectiveness of activated carbon R95 respirators in reducing the inhalation of combustion by-products in Hanoi, Vietnam: a demonstration study

**DOI:** 10.1186/1476-069X-11-72

**Published:** 2012-09-26

**Authors:** Heiman FL Wertheim, Dang Minh Ngoc, Marcel Wolbers, Ta Thi Binh, Nguyễn Thị Thanh Hải, Nguyễn Quỳnh Loan, Phạm Thanh Tú, Andreas Sjodin, Lovisa Romanoff, Zheng Li, Jochen F Mueller, Karen Kennedy, Jeremy Farrar, Kasia Stepniewska, Peter Horby, Annette Fox, Nguyen Duy Bao

**Affiliations:** 1 Oxford University Clinical Research Unit and Wellcome Trust Major Overseas Programme, Hanoi, Vietnam; 2 Nuffield Department of Clinical Medicine, Oxford, United Kingdom; 3 National Institute of Occupational and Environmental Health, Hanoi, Vietnam; 4 Centers for Disease Control and Prevention, Atlanta, USA; 5 The University of Queensland, The National Research Center for Toxicology (Entox), Queensland, Australia; 6 Mahidol-Oxford Tropical Medicine Research Unit, Mahidol University, Bangkok, Thailand

**Keywords:** Urine, Pollution, Urban, Polycyclic aromatic hydrocarbons, PAH, 1-hydroxypyrene, Respirator

## Abstract

**Background:**

Urban air pollution is an increasing health problem, particularly in Asia, where the combustion of fossil fuels has increased rapidly as a result of industrialization and socio-economic development. The adverse health impacts of urban air pollution are well established, but less is known about effective intervention strategies. In this demonstration study we set out to establish methods to assess whether wearing an R95 activated carbon respirator could reduce intake of polycyclic aromatic hydrocarbons (PAH) in street workers in Hanoi, Vietnam.

**Methods:**

In this demonstration study we performed a cross-over study in which non-smoking participants that worked at least 4 hours per day on the street in Hanoi were randomly allocated to specific respirator wearing sequences for a duration of 2 weeks. Urines were collected after each period, i.e. twice per week, at the end of the working day to measure hydroxy PAHs (OH-PAH) using gas chromatography/high resolution mass spectrometry. The primary endpoint was the urinary concentration of 1-hydroxypyrene (1-OHP).

**Results:**

Forty-four participants (54.5% male, median age 40 years) were enrolled with the majority being motorbike taxi drivers (38.6%) or street vendors (34.1%). The baseline creatinine corrected urinary level for 1-OHP was much higher than other international comparisons: 1020 ng/g creatinine (IQR: 604–1551). Wearing a R95 mask had no significant effect on 1-OHP levels: estimated multiplicative effect 1.0 (95% CI: 0.92-1.09) or other OH-PAHs, except 1-hydroxynaphthalene (1-OHN): 0.86 (95% CI: 0.11-0.96).

**Conclusions:**

High levels of urine OH-PAHs were found in Hanoi street workers. No effect was seen on urine OH-PAH levels by wearing R95 particulate respirators in an area of high urban air pollution, except for 1-OHN. A lack of effect may be de to gaseous phase PAHs that were not filtered efficiently by the respirator. The high levels of urinary OH-PAHs found, urges for effective interventions.

**Trial registration:**

ISRCTN74390617 (date of assignation: 04/08/2009).

## Background

Urban air pollution is an increasing health problem in Asia, where the combustion of fossil fuels has increased rapidly as a result of industrialization and socio-economic development [1-5]. Combustion produces a complex mixture of pollutants consisting of primary emissions and products of atmospheric transformation [4,6]. It is estimated that air pollution in urban areas worldwide causes approximately 3% of adult cardiopulmonary disease deaths approximately 5% of mortality from malignancies of the respiratory tract, and about 1% of mortality from acute respiratory illness (ARI) in children [4]. This results in 0.80 million premature deaths with 6.4 million years of life lost (YLL) and occurs predominantly in developing countries, with 20%-39% of attributable YLL in the South East Asia Region [4]. To date, most studies on health and urban air pollution have been performed in developed countries, where pollution levels are generally within the limits considered by WHO to be safe [6]. It is essential to study the health impacts of air pollution in the large cities of Asia where air pollution levels exceed WHO standards and to develop interventions to reduce the burden of associated morbidity and mortality.

According to the Global Environment Outlook released by the United Nations Environment Program in October 2007, both Hanoi and Ho Chi Minh City, Vietnam, have serious problems with air pollution, with pollution parameters exceeding WHO standards [7]. The Vietnamese Ministry of Natural Resources and Environment (MoNRE) estimated that traffic emissions are responsible for 70% of all air pollution, 85% of carbon monoxide (CO) and 96% of volatile organic compounds [7]. The levels of respirable particulate matter, with a particle size of < 10 μm (PM_10_), in 2004 in several Hanoi districts exceeded the limits of the WHO threshold of 50 μg/m^3^ up to five fold [7]. Since that study there has been further rapid urbanization in Hanoi and massive expansion in the number of motor vehicles. One study in Vietnam measured atmospheric polycyclic aromatic hydrocarbons (PAHs) at 10 different roadside sites in Hanoi in August 2005 [8]. The concentrations reported are significantly higher than those from other countries, often exceeding recommended maximum thresholds by the WHO. The measured atmospheric PAHs mainly originated from motorcycles without catalytic converters [8].

Long-term exposure to urban air pollution has been associated with mutagenesis and cancer whereas short-term exposure has been associated with pulmonary inflammation, increased susceptibility to bacterial and viral infections, allergens and increased susceptibility to lung damage [9-11]. Current evidence of an impact on human health has been mostly indirect and comes largely from ecological studies [9,12,13], which although well conducted, do not provide evidence for causal associations. Limited cohort studies provide more direct evidence but still have not been able to establish a causal relationship [14].

Urban air pollution is likely to increase, especially in the Asian region, where urban air pollution contributes to 500,000 premature deaths annually [7]. Interventions to prevent disease and mortality in regions with high urban air pollution levels are urgently required. Combustion of gasoline and diesel fuels releases particulates and PAHs and in Hanoi motor vehicles have been the major source of PAHs [15]. In this demonstration study we set out to determine the PAH intake of workers in high traffic areas of Hanoi by measuring PAH metabolites (OH-PAHs) in the urine and to pilot whether the PAH intake can be reduced by wearing a particulate respirator that can filter particulate bound PAHs [16-19]. Particulate respirators (e.g. N95 or R95) that can efficiently filter particulate matter, have shown to reduce OH-PAHs in industrial settings, however have never been tested in a community based setting with high PAH levels [20-22].

## Methods

### Study site and study population

This demonstration study was conducted by the National Institute of Occupational and Environmental Health (NIOEH) in Hanoi, Vietnam. The target population was healthy adult volunteers that worked outside on the streets of Hanoi for at least 4 hours per day. Exclusion criteria include other PAH exposures that may lead to increased OH-PAHs levels in the urine: smoking tobacco, cooking at home with biomass fuel, unwilling to stop eating grilled food during study period. Also failing the respirator mask fit test was an exclusion criterion. Institutional Review Board approval was obtained from Oxford University Tropical Research Ethics Committee (OXTREC) and NIOEH, and was added to the trial registry under number ISRCTN74390617. Written informed consent was obtained from all participants.

### Study intervention and design

The intervention consisted of wearing a R95 particulate respirator (9913 K respirator, 3 M, USA) with a filtration efficiency of at least 95% when tested with 0.3 mm dioctyl phthalate aerosols. The respirator has activated carbon to reduce nuisance levels of odours, and has an adjustable nose clip for better fit and for preventing fogging of eye wear. The activated carbon has been shown to be able to filter vapours, like toluene and benzene, with a 10% breakthrough value of at least 60 minutes [23]. All participants were instructed on how to wear the mask and were required to pass the respirator ‘fit test’ using the ‘saccharin solution aerosol protocol’ according to the Occupational Safety & Health Administration (USA) guidelines [24].

Urine 1-hydroxypryrene (1-OHP) concentration was chosen as the primary endpoint in this study because it is the metabolite of pyrene, a common component of PAH mixtures, and is a good biological indicator of PAH exposure [15,25-27]. The median half-life of urine 1-OHP was estimated recently to be 3.9 hours, but can vary and be up to 18 h depending on the exposure [25,28]. We used a cross-over study design to study the effect of wearing R95 respirators in which participants were randomly allocated to specific respirator wearing sequences: ABBA or BAAB, where ‘A’ is wearing the respirator for two consecutive days during working hours and ‘B’ is not wearing the respirator for two consecutive days (Table 1). We chose two days as previous work showed that OH-PAHs return to their baseline levels within 24–48 hours after the exposure [28]. A new respirator was provided for each day a mask was to be worn. Compliance was assessed through weekly unannounced visits and checks by study team members. Furthermore, reminder text messages were sent to the mobile phones of the participants when they needed to wear the mask. Participants were asked to note in their diary whether they wore the mask or not. 

**Table 1:** Study profile

**Day**	**Group ABBA**	**Group BAAB**
*Friday*	Evening urine	Evening urine
*Weekend*	No intervention	No intervention
*Monday*	Wear respirator	No respirator
*Tuesday*	Wear respirator, evening urine	No respirator, evening urine
*Wednesday*	No intervention	No intervention
*Thursday*	No respirator	Wear respirator
*Friday*	No respirator, evening urine	Wear respirator, evening urine
*Weekend*	No intervention	No intervention
*Monday*	No respirator	Wear respirator
*Tuesday*	No respirator, evening urine	Wear respirator, evening urine
*Wednesday*	No intervention	No intervention
*Thursday*	Wear respirator	No respirator
*Friday*	Wear respirator, evening urine	No respirator, evening urine

### Data and urine specimen collection

The following data were collected from the participants: age, sex, occupation, medical history, weight, smoking behavior, living conditions, and prior use of a face mask unrelated to the study. A daily diary was kept with the following assessments: respiratory symptoms (sneezing, nasal congestion, nasal discharge, throat pain, cough, dyspnea, eye irritation, other), respirator comfort, food consumed, time start/stop working, and distance driven on motorbike. Urine specimens (10 to 20 mL) were collected on Tuesday and Friday afternoon by the participant in a labeled sterile container (Table 1). The urine specimens were immediately transported on ice and frozen at −80°C until testing.

### OH-PAH analysis in urine

At the end of the study, collected urine samples were shipped on dry ice to the Centers for Disease Control and Prevention (CDC), Atlanta, USA for analysis of OH-PAHs using gas chromatography/high resolution mass spectrometry (GC-HRMS) according to previous described methods [29]. The methodology for measuring OH-PAH metabolites, present in human urine as glucuronide and/or sulfate conjugates, is based on enzymatic deconjugation of the analytes to yield free OH-PAHs, followed by automatic liquid-liquid extraction into pentene using the Gilson 215 Liquid Handler (Gilson Inc., Middleton, WI). The sample extracts are thereafter evaporated under a chemical fume hood to remove the pentene solvent. Finally, the extracts are re-constituted in toluene, derivatized to yield the trimethylsiloxane derivatives. Analytical determination of the target analytes were performed by gas chromatography isotope dilution high resolution mass spectrometry (GC-IDHRMS) employing a MAT95XP (ThermoFinnigan MAT, Bremen, Germany) instrument. Creatinine concentration was measured to control for the effect of fluid intake and loss and adjust OH-PAH concentrations accordingly.

### Cotinine urine analysis

Cotinine concentrations were measured at NIOEH to detect participants who were exposed to high levels of tobacco smoke. The urine samples (5 mL) were added to sodium hydroxide (5 M, 15 mL) and antifoam agent (Phenol Red, 5% w/w, 1 mL) and extracted with dichloromethane. After mixing by vortex and separation of the phases by centrifugation, the organic phase was transferred to a glass tube and the volume of the solvent was evaporated. The residue was dissolved in 1 mL toluene from which a 2 μl splitless injection was made on a Hewlett Packard model HP5890A gas chromatograph (Agilent Technologies, Atlanta, GA, USA), equipped with an Nitrogen Phosphorus Detector (NPD).

### Sample size calculation

For the sample size calculation we used the previously reported concentrations of pyrene in air for Hanoi [8], a half life of 1-OHP of 3.9 hours (see also Table 2) [28], and we assumed that the participants are compliant in wearing their masks during their work. The total inhaled volume over 8 hours was estimated to be 2880 L, the daily urinary excretion 2 L, and the pyrene intake to range from 104 to 789 ng/8 hours. Assuming that the R95 mask reduces PAH intake by 90%, that normal pyrene intake is minimal (i.e. 104 ng) and that the baseline 1-OHP level is 30 ng/L, then urinary 1-OHP will be 82 ng/L (104 ng/2 L + 30 ng/L) without a respirator and 35.2 ng/L ([104 ng × 0.1]/2 L + 30 ng/L) with a respirator. Assuming that the within-subject standard deviation of a measurement is of similar size as the respirator effect (corresponding to 0.85 on the natural log-scale) and a two-sided alpha level of 0.05, a total of 24 subjects would attain a power of 90% for a two-period cross-over trial and even higher for our 4-period design. Due to expected large variations in dietary intake and other possible exposures we decided to enroll 44 subjects. 

**Table 2:** Baseline OH-PAHs levels (ng/g creatinine) and OH-PAHs’ half-lives from reference 27

**OH-PAH**	**Median half-life t 1/2,from ref 27**	**Size**	**Median ng/g creat**	IQR*
*1-hydroxynaphthalene*	4.3	44	6172	4297 - 11220
*1-hydroxyphenanthrene*	5.1	44	562	418 - 839
*1-hydroxypyrene*	3.9	44	1020	604 - 1551
*2-hydroxyfluorene*	2.9	44	811	575 - 1106
*2-hydroxynaphthalene*	2.5	44	7051	5138 - 11820
*2-hydroxyphenanthrene*	3.9	44	358	254 - 534
*3-hydroxyfluororene*	6.1	44	284	162 - 450
*3-hydroxyphenanthrene*	4.1	44	475	288 - 833
*4-hydroxyphenanthrene*	3.5	44	102	75 - 146
*9-hydroxyfluorene*	3.1	44	1400	1018 - 1822

*IQR: interquartile range.

### Statistical analysis

An overall respirator effect on the urine concentration of each measured metabolite was derived with a linear mixed effect model which modeled follow-up measurements as depending on the current intervention in a period (respirator versus no respirator), the period (4 periods in total) and a random subject effect (to account for correlation of measurements within an individual). The analysis was performed on all enrolled individuals and repeated on the per protocol population after excluding subjects with positive cotinine at baseline as this suggested active smoking or exposure to second hand smoke, and periods where participants were non-compliant or compliance status unknown. An adjusted analysis was done where the model was additionally adjusted for age, gender, occupation, and the baseline value of the metabolite. All metabolites were log-transformed prior to the analysis but mask effects were back-transformed to the original scale after analysis, i.e. reported effects are on the multiplicative scale. We also tested for carry-over, i.e. mask effect-period interactions. All analyses were performed with the free software R version 2.11.1 (R Foundation for Statistical Computing, Vienna, Austria).

## Results

**Figure 1:**
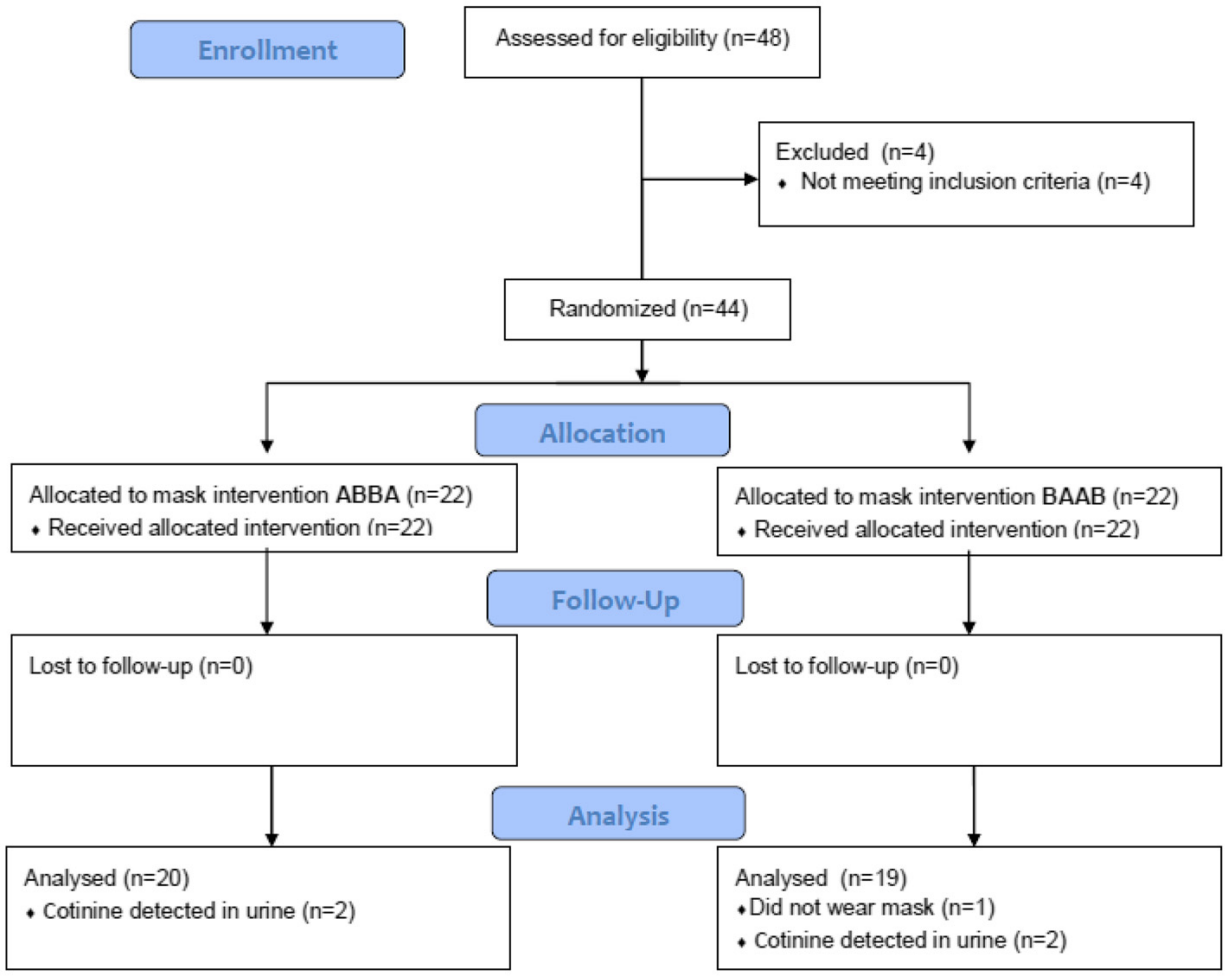
CONSORT flow diagram.

Forty-four participants (54.5% male, median age 40 years) were enrolled into the study from May to June, 2009 (see Table 3 for baseline characteristics and Figure 1 for CONSORT flow diagram). The majority of the participants were motorbike drivers (38.6%) or street vendors (36.5%). Other occupations were: security (6.8%), bike repair (4.5%), and other work that was done on the street (13.6%). None of the participants were known to have chronic lung disease. Most participants lived close to their work place (median: 0.5 km, IQR: 0.1-1.0 km). During the day the participants travelled a median of 6 km per day (inter quartile range [IQR]: 2–100 km) and spent a median of 10 hours (IQR: 8–12 hours) hours outside on the street. The mean average monthly wage was 1.6 million Vietnam dong (approximately 85 US$). In Vietnamese cities it is common to wear a cloth mask on the street. Before the study 40.9% never wore a mask, 27.3% sometimes, and 31.8% often. Most participants (88.6%) complained that the R95 respirator was not comfortable. Other complaints were that the mask was too tight (n = 2), painful (n = 2), or hard to breath through (n = 1). Table 2 lists the median baseline urinary concentrations of OH-PAHs of all participants.

**Table 3:** Baseline characteristics

	**ABBA group (n =22)**	**BAAB group (n =22)**	**Total (n = 44)**
Sex (n,%)			
*female*	12 (54.5%)	8 (36.4%)	20 (45.5%)
*male*	10 (45.5%)	14 (63.6%)	24 (54.5%)
Age (median, IQR)	42.5 (35–55)	39 (29–50)	40 (31–51)
Occupation (n,%)			
*motorbike driver*	6 (27.3%)	11 (50.0%)	17 (38.6%)
*street vendor*	9 (40.9%)	6 (27.3%)	13 (34.1%)
*other*	7 (31.8%)	5 (22.7%)	12 (27.3%)

Other occupations were: security, bike repair, and other street-related work.

### Evaluation of mask effect

**Figure 2:**
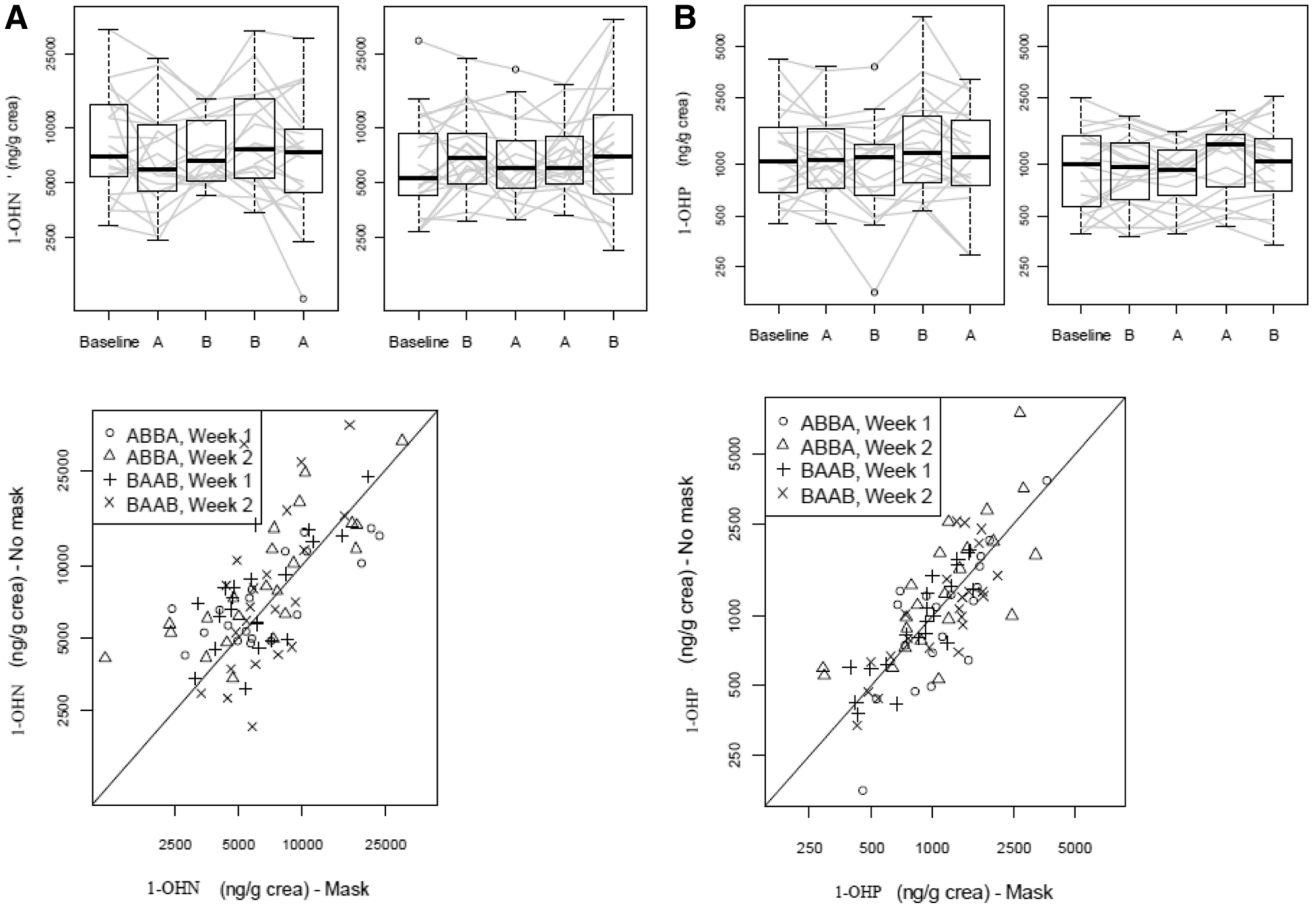
**Figures for 1-OHN (panelA) and 1-OHP (panelB) line plots ofvalues for each patientwith overlaid boxplots andscatterplots of values withmask versus those withoutmask (if values withoutmask are higher, mostpoints of the scatterplotshould be above thediagonal). A**: wore study mask; **B**: did not wear study mask.

During respirator periods, the study mask was worn for a median of 7 hours per day (IQR: 6.5-8). One participant wore the mask on one study day only for just 3 hours as the mask was considered too tight and the participant found it difficult to breath. One individual need to be corrected in how to wear the mask as she was wearing it improperly during a visit. Few follow-up measurements were missing. The only metabolites with >1 of the 176 follow-up measurements missing were 1-hydroxynaphthalene (1-OHN; 4, 2.27%), 1-OHP (4, 2.27%), and 9-hydroxyfluorene (6, 3.41%). There was no evidence that wearing a R95 respirator affected the primary endpoint of the urinary concentration of 1-OHP (Table 4, Figure 2). Moreover, the lower confidence limit for the mask effect excluded an average lowering of 1-OHP concentration due to mask wearing by more than 8%. Results for the adjusted analysis and per protocol analysis (n = 39 as cotinine was detected in the urine of 4 volunteers and one volunteer had unknown compliance status; moreover, 3 periods with unknown compliance or non-compliance were excluded) were consistent with the main analysis (Table 4). There was also no evidence of a carry-over effect (p = 0.42); however, there was a significant period effect (p < 0.001), which was mainly due to higher measurements in period 3 in both groups (Figure 2). There was also no evidence for a mask effect for any of the other metabolites except for 1-OHN. For 1-OHN, the measurements with mask were on average by a factor of 0.86 (95% CI 0.77 to 0.96) lower.

**Table 4:** Effect of mask wearing on PAH metabolites (multiplicative scale)

**PAH metabolite**	**Mask effect**	**Mask effect (adjusted)**	**Mask effect (per protocol)**	**Test for period effect**	**Test for carry-over**
1-hydroxynapthalene	0.86 (0.77,0.96); p = 0.007	0.86 (0.77,0.96); p = 0.007	0.87 (0.77,0.98); p = 0.02	p = 0.38	p = 0.85
2-hydroxynapthalene	0.95 (0.84,1.08); p = 0.42	0.95 (0.84,1.08);p = 0.47	0.96 (0.83,1.1); p = 0.54	p = 0.41	p = 0.98
2-hydroxyfluorene	0.98 (0.9,1.07); p = 0.61	0.98 (0.9,10.07); p = 0.66	0.97 (0.88,1.07); p = 0.58	p = 0.34	p = 0.56
3-hydroxyfluorene	0.97 (0.88, 1.06); p = 0.48	0.97 (0.88, 1.06); p = 0.5	0.97 (0.87, 1.08); p = 0.58	p = 0.05	p = 0.6
9-hydroxyfluorene	0.94 (0.85, 1.04); p = 0.23	0.94 (0.85, 1.04); p = 0.22	0.95 (0.85, 1.06); p = 0.33	p < 0.001	p = 0.59
1-hydroxyphenanthrene	0.97 (0.89, 1.07); p = 0.58	0.98 (0.89, 1.08); p = 0.66	0.98 (0.88, 1.08); p = 0.65	p = 0.11	p-0.59
2-hydroxyphenanthrene	1.02 (0.92, 1.12); p = 0.72	1.02 (0.93, 1.13); p = 0.67	1.02 (0.91, 1.14); p = 0.74	p = 0.45	p = 0.15
3-hydroxyphenanthrene	1.01 (0.92, 1.1); p = 0.88	1.01 (0.92, 1.11); p = 0.85	1.03 (0.93, 1.14); p = 0.6	p = 0.69	p = 0.63
4-hydroxyphenanthrene	0.96 (0.86, 1.08); p = 0.53	0.97 (0.86, 1.09); p = 0.59	0.97 (0.86, 1.1); p = 0.66	p = 0.01	p = 0.46
1-hydroxyprene	1.00 (0.92, 1.09); p = 0.98	1.00 (0.92, 1.09); p = 0.96	1.01 (0.92, 1.11); p = 0.84	p < 0.001	p = 0.42

Effects are displayed as estimate (95% confidence interval); p-value.

## Discussion

Our data show that the overall levels of PAH metabolites in the urine of Hanoi street workers were very high, indicating that there is high exposure to PAHs in Hanoi (Table 2 and 5). In Table 5 the median level of 1-OHP in other non-occupational populations are provided and compared to our Hanoi data, showing that levels were for example 24-fold higher than a studied USA population. The levels found are not as high as those found in workers in the petrochemical industry, particularly coke-oven workers [30]. These data, together with the known high levels of PAHs in ambient air in Hanoi, suggest that inhalation is the main route of exposure. Despite this, in this study with a relative small sample size we were not able to demonstrate an effect of the mask on urine concentrations of OH-PAHs. 

**Table 5:** **Comparison of urinary concentrations (ng/g) of 1-OHP in Hanoi to other non-occupational populations reported in the literature (modified from Li Z., et al. 2010**[31]**)**

**Population**	**Country**	**Age group**	**Size**	**Median 1-OHP level (5-95thpercentile)**
*Adults*	USA	≥ 20	1625	41 (15–233)
*Students*	Korea	mean 23	129	58 (19–212)
*Adults*	Germany	≥ 20	495	88 (22–362)
*Adults*	Canada	na	140	174 (39–617)
*Adults*	Italy	22 - 81	327	149 (52–654)
*Street workers (this study)*	Vietnam	29 - 55	44	1020 (416–3453)

With the exception of 1-OHN, no effect was seen on urine OH-PAH levels by wearing the study mask. In the case of 1-OHN, inhalation would be the overall main route of exposure [31]. However, for 2-OHN no effect was seen, indicating this may be a chance finding as ten metabolites were analyzed in this study. As urinary OH-PAH measures total PAH exposure, other sources of exposure need to be considered that may explain the lack of effect of the study mask. Possible explanations are: (1) high exposure to vapour phase PAHs that are not filtered efficiently by the mask [8,23], (2) high PAH exposure outside working hours when not wearing the mask, (3) masks were not worn for a sufficient number of consecutive days to allow for sufficient clearance time for inhaled PAHs to be excreted by the urine at the end of the mask-wearing period or non-compliance or improper fit, (4) other exposure sources such as dermal and ingestion exposure, and (5) the mask was only tested in a short time period in a single season, which may affect the results.

The average concentrations of 47 PAHs in Hanoi in 2005 were: 63 ± 82 ngm^-3^ in particulate matter and 480 ± 300 ngm^-3^ in the vapour phase [8]. Also the PAHs of which we assessed the metabolites in the urine were more abundant in vapour phase, than in particulate matter. High molecular weight PAHs are predominantly adsorbed on soot and particulate matter and the amount present in the vapor phase is dependent upon the specific PAH and other properties such as temperature and concentration [32]. The PAH levels in particulate matter and vapour-phase in Hanoi at the time of the study was not assessed. This is an important limitation of the study because the R95 respirator used was only designed to filter out particulate matter and not vapour-phase PAHs. Though the masks did have activated carbon, this would not be sufficient to filter out vapour-phase PAHs [23].

It is highly possible that the participants were exposed to high levels of PAHs when they were not wearing the mask. This is because the participants lived close to their working place and according to the study protocol they were only required to wear the mask during working hours. Data from the Swiss-Vietnam Clean Air Program show that pollution is heaviest at the roadside of main roads where our participants typically worked and less in the residential areas (Swiss-Vietnam Clean Air Program, unpublished results). Still, the exposure during off-work hours is likely to be high, which may have had an impact on the measured OH-PAHs levels in the urine. Furthermore, non-compliance or improper mask fits may have influenced the results. This study was performed in a non-regulated setting where it is difficult to monitor compliance. However, visits by study staff did show that the masks were worn properly, except for one case.

Other potential sources of PAHs that could have influenced our results include: grilled food, cigarette smoke, and dermal exposure. With questionnaires, the volunteers were asked about these potential sources and cotinine levels were measured. In four cases we detected cotinine in the urine and excluded them from the per-protocol analysis, but this did not alter the results. No subject reported eating barbecued or grilled food during the study period. Significant dermal exposure in people exposed to urban ambient air pollution is unlikely, as this is generally considered a risk in those working in particular industries with dermal exposure to oil compounds [33].

As stated earlier, we cannot rule out, that subjects were exposed to significant levels of PAHs at home and while sleeping, and therefore, the study subjects may have had limited time for clearance of PAH exposure from the body between their work shifts. Furthermore, the two days of wearing the respirator may not have been sufficient, since the half-lives of PAHs vary in different reports and can exceed 24 hours [25]. Furthermore, the two days may also not be sufficient for clearance as the OH-PAH exposure of our participants was not a single exposure, but continuous [28]. Ingestion exposures are reportedly lower than dermal or inhalation exposures [28].

As far as we know this is the first randomized intervention study using respirators to reduce exposure to PAHs in a non-occupational community setting. Most studies are performed in occupational settings for which good guidelines for intervention study designs have been developed (see also: http://www.aiha.org/insideaiha/volunteergroups/RPC/Documents/rpc-terms.pdf) [34]. One study in 1996, assessed PAH biomarkers in urine in 89 Bangkok police officers working in traffic. This study did report a reduction in biomarkers by wearing a simple face masks while they were on duty for a week [35]. Other studies using particulate respirators in occupational settings showed a reduction in OH-PAH levels in the urine [20-22]. None of these studies had a randomized design. These studies state that the masks protect against the PAH bound to particulate matters as reported in another study by Alexandrie et al. [36].

One of the key differences with this study is that in all studies the workers wore the masks for a longer time period. It is recommended that in subsequent studies the mask should be worn for a longer consecutive period and preferably in various climate conditions that cause variation in pollution levels. Furthermore, respirators that can also filter vapour phase PAHs need to be considered in future studies in this setting. The only qualitative measure we had that did show a mask effect was that some motorbike drivers anecdotally reported that when they wore the study mask they had less respiratory symptoms after a day of work. But this information was not supported by what was reported in the diaries.

## Conclusions

The concentrations of OH-PAHs in the urine (pollution biomarkers) were extremely high in Hanoi street workers. These high levels warrant further intervention studies to mitigate the health risks related to air pollution. In this demonstration study, no effect was seen on urine OH-PAH levels by wearing R95 masks during work hours for two days in Hanoi, except for 1-OHN. It is recommended that in subsequent studies the mask should be worn for a longer consecutive period and preferably in various climate conditions that cause variation in pollution levels. Furthermore, respirators that can also filter vapour phase PAHs need to be considered in future studies in this setting.

## Abbreviations

ARI: Acute Respiratory Illness; CDC: Centers for Disease Control and Prevention; CI: Confidence Interval; CO: Carbon Monoxide; GC-HRMS: Gas Chromatography/High Resolution Mass Spectrometry; GC-IDHRMS: Gas Chromatography Isotope Dilution High Resolution Mass Spectrometry; IQR: Inter Quartile Range; NIOEH: National Institute of Occupational and Environmental Health; OH-PAH: Hydroxy Polycyclic Aromatic Hydrocarbons; 1-OHN: 1-hydroxynaphthalene; 1-OHP: 1-hydroxypyrene; PAH: Polycyclic Aromatic Hydrocarbons; WHO: World Health Organization; YLL: Years of Life Lost.

## Competing interests

Financial competing interests: The R95 respirators were supplied free of charge by 3 M Inc. for this study. It was an initiative of the study team to ask 3 M to supply the masks without charge and to provide training to study participants on how to wear the mask and perform the mask fit test. 3 M had no role in the study design, study execution, data analysis or report writing. The authors declare that they have no further competing interests. Non-financial competing interests: none declared.

## Authors’ contributions

HW: conceived the study, study design, coordination, analysis, write manuscript; DN, JM, AF, PH, ZL, AS, NB: study design, analysis, helped draft paper; DN, LR, AS, JM, ZL, KK: carried pollution assessments, analysis, and helped draft paper; KS: study design; MW: study design, statistical analysis and draft paper; TB, NH, NL, PT: study design, enroll study participants, follow up participants, collect and store specimens. All authors read and approved the final manuscript.
